# Shoes

**Published:** 1878-02

**Authors:** 


### SHOES.

The feet are the great avenues of death to multitudes every year ; cold feet, damp feet, wet feet give colds which settle on the lungs and light up the fires of consumption which burn away until nothing is left but skin and bone, and the poor body falls into the grave, hence the importance of clothing the feet properly. More than two thousand years ago the Jews made shoes of leather and wood while their soldiers sometimes formed them of brass and iron ; the Egyptians used papyrus ; the Chinese wore shoes made of silk, leather, rushes, iron, brass, wood, bark, gold and silver. The Greeks and Romans used leather, reaching generally to the middle of the leg, sometimes however using only enough leather to cover the sole of the foot, black shoes were worn by ordinary persons, of rank, the women wore white, but on ceremonial days, the magistrates wore red shoes. The ingenuity of modern times has not as yet furnished a convenient, comfortable and healthful covering for the feet; the requisites are pliancy so as to allow the toes, nails and joints to maintain their perfectly natural condition, which so greatly promotes easy and healthful walking; the next necessity is that the soles shall be impervious from dampness without and dampness from within; there should be ample ventilation, otherwise the gases and perspiration constantly escaping from the feet, as well as from every part of the human body will condense, not only causing an unhealthful dampness, but so confining the odors as to sensibly affect the atmosphere of a large room, the moment the covering is removed. The shoe itself should not come above the ankle, because, first, the emanations would readily escape, and fresh air be constantly admitted; second, because the ankle, being unsupported, would by use, more rely upon itself, would become daily stronger, more supple and more elastic, with greatly less danger of dislocation; hence the wearing of boots habitually is a gross absurdity, a relic of barbarism. The sole of the shoe should be painted several times with castor oil, being allowed to dry most thoroughly between each application; this would take the disagreable creak out of shoes and would keep out the water very effectually without the necessity of having a cumbrously thick sole. The upper part of the shoe should be made of canvass, of a color suited to the weather and to the habits of the individual, fitting but moderatly tight. The feet should be placed in a basin of cold water every morning for a few seconds, just deep enough to cover the toes; wipe dry, dress and walk off. Once or twice a week the feet should be held in water, made comfortably warm, for some ten minutes, adding hotter water from time to time, using a little soap; if at the end of this bathing at night the feet were placed in a pan of cold water, toe-deep for less than a quarter of a minute, it would greatly aid in giving tone to the skin, vigor to the circulation and softness to the skin, and thus do much towards keeping them comfortably warm. A tablespoonful of Chloride of lime in a basin of warm water is an excellent wash for removing foot odor: before retiring to bed most especially in fire time of year, hold both feet before a blazing fire, stockings removed, for ten minutes at least, rubbing them with the hands all the time until they feel perfectly dry and warm; such a process will warm the feet more effectually in five minutes than can be done in an hour by holding them to the fire with stockings and shoes on.

